# H∞ Robust Control of a Large-Piston MEMS Micromirror for Compact Fourier Transform Spectrometer Systems

**DOI:** 10.3390/s18020508

**Published:** 2018-02-08

**Authors:** Huipeng Chen, Mengyuan Li, Yi Zhang, Huikai Xie, Chang Chen, Zhangming Peng, Shaohui Su

**Affiliations:** 1 School of Mechanical Engineering, HangZhou DianZi University, Hangzhou 310018, China; hpchen@hdu.edu.cn (H.C.); chenchang@hdu.edu.cn (C.C.); pzm@hdu.edu.cn (Z.P.); 2 Department of Electrical and Computer Engineering, University of Florida, Gainesville, FL 32611, USA; limengyuan@ufl.edu (M.L.); hkxie@ece.ufl.edu (H.X.); 3 College of Mechanical and Electronic Engineering, Shangdong University of Science and Technology, Qingdao 266590, China; zhangyi@sdust.edu.cn; 4 School of Information and Electronics, Beijing Institute of Technology, Beijing 100081, China; ytd@bit.edu.cn

**Keywords:** electrothermal micromirror, robust control, bimorph actuator modeling, active tilting rejection, Fourier transform spectrometer

## Abstract

Incorporating linear-scanning micro-electro-mechanical systems (MEMS) micromirrors into Fourier transform spectral acquisition systems can greatly reduce the size of the spectrometer equipment, making portable Fourier transform spectrometers (FTS) possible. How to minimize the tilting of the MEMS mirror plate during its large linear scan is a major problem in this application. In this work, an FTS system has been constructed based on a biaxial MEMS micromirror with a large-piston displacement of 180 μm, and a biaxial H∞ robust controller is designed. Compared with open-loop control and proportional-integral-derivative (PID) closed-loop control, H∞ robust control has good stability and robustness. The experimental results show that the stable scanning displacement reaches 110.9 μm under the H∞ robust control, and the tilting angle of the MEMS mirror plate in that full scanning range falls within ±0.0014°. Without control, the FTS system cannot generate meaningful spectra. In contrast, the FTS yields a clean spectrum with a full width at half maximum (FWHM) spectral linewidth of 96 cm^−1^ under the H∞ robust control. Moreover, the FTS system can maintain good stability and robustness under various driving conditions.

## 1. Introduction

Fourier transform infrared spectroscopy (FTIR) [1] is a technique that is used to obtain absorption or emission infrared (IR) spectra of various matters and determine materials’ compositions and concentrations in both laboratory and field environments. A Fourier transform spectrometer (FTS) is based on a Michelson interferometer consisting of a beam splitter, a photodetector (PD), and one movable mirror and one fixed mirror, respectively, in its two optical path arms. Conventional FTS systems are only for lab use, as they are expensive and bulky largely due to the complex scanning mirror system [1].

Recently, FTS systems based on micro-electro-mechanical system (MEMS) micromirrors begin to emerge, and such miniature FTS systems can enable real-time, in-field analysis in many environments such as national border checkpoints and in natural or manmade hazardous conditions [2,3]. For miniature FTS systems, the scanning characteristics of the moving MEMS micromirror are critical. Electrothermal micromirrors are more widely used in FTS than other types, such as piezoelectric, electromagnetic, and electrostatic micromirrors, because they can provide much larger linear scan range at low drive voltage [4,5,6,7,8,9,10].

In principle [11], for a FTS, the Fourier Transform of the interferogram yields a spectrum whose resolvable spectral linewidth is inversely proportional to the movable mirror’s scan range, i.e., the larger the linear scan range of the movable mirror, the higher the achievable spectral resolution is. However, limited by the fabrication process variations, the piston motion of MEMS micromirrors always comes with tilting. The tilting during the mirror’s piston deteriorates the interferogram, resulting in low spectral resolution or even no recoverable spectrum. Thus, controlling the mirror’s tilting has become the biggest challenge in practical, MEMS-based FTS systems.

Several tilt control methods have been developed to reduce the tilting of the movable micromirror of the FTS system during scanning motion. Wu et al. [12] developed an FTS based on the dual-reflective MEMS mirror. When the driving signal was not compensated or controlled, the tilting angle was 0.7° during the scan. By using a pre-shaped drive signal, the mirror tilting was reduced to 0.06°. S. R. Samuelson et al. [13] proposed a piston motion micromirror with a laddered inverted-series-connected (ISC) electrothermal actuator array and demonstrated an uncompensated tilt of 0.25° over its full displacement range. By designing a pair of ratio optimized drive signals, the tilting angle was reduced to 0.004° [14]. Wang et al. [11] reported a large-stroke electrothermal MEMS mirror with an original tilting angle 0.3°. In order to reduce the tilting angle, the micromirror was driven by an open-loop control using a pair of corrected ramp drive signals, and the final tilting angle was reduced to ±0.002°.

In view of the high sensitivity of open loop control to the environmental variations and disturbances, there have been numerous studies on the design of closed-loop controllers for improving the repeatability and stability of micromirrors [15,16,17], most of which are focused on electrostatic micromirrors. A study of closed-loop tilt control for a single-axis electrothermal micromirror has just recently been reported [18], in which the tilt angle was controlled within ±0.0015°. Although the closed-loop control algorithm developed in this study improved the robustness of the electrothermal micromirror scanning, there are still some practical problems that have not been considered and solved. First of all, the uniaxial electrothermal micromirror previously studied has no actuators in the orthogonal axis, and thus the jitter in the orthogonal axis is in an uncontrolled state. Secondly, the change of the characteristics of the electrothermal micromirror due to aging and any changes in the operating environment may cause the originally stable system to lose its stability. 

In this work, a dual-axis electrothermal micromirror instead of a uniaxial micromirror is employed in the MEMS FTS system. Thus, both directions of the micromirror can be controlled to avoid the instability caused by no control in one axis. The coupling relationship between the input and output of the micromirror system has been studied and analyzed, which lays a foundation for the independent control design of the *x* and *y* direction. In order to improve the robustness of the MEMS FTS system, an H∞ robust control method is proposed and the H∞ controller is designed. As a comparison, a proportional-integral-derivative (PID) controller and a look-up table controller are also implemented. The experimental results show that with the H∞ control, not only the titling angle is greatly reduced, but also it has the advantage of good anti-disturbance ability. 

This paper is organized as follows. Section 2 introduces the electrothermal MEMS mirror and its experimental model. In Section 3, the design of an H∞ robust controller is introduced in detail, and a PID controller and a look-up table driving curve are also presented. In Section 4, the experimental results with the H∞ robust controller, PID controller, and look-up table driving curve on the MEMS mirror and their application in the MEMS FTS system are analyzed and compared.

## 2. The Electrothermal Micromirror

### 2.1. Device Description

**Figure 1:**
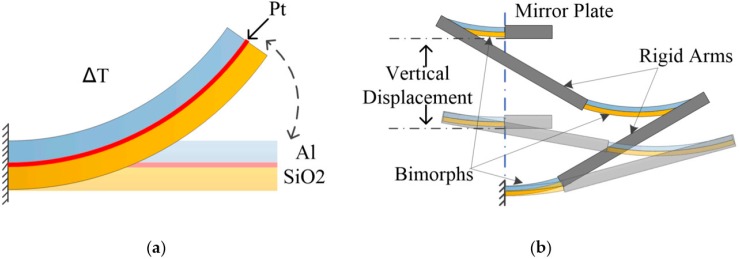
(**a**) Bimorph structure, ∆T is the temperature change of the bimorph; (**b**) large displacement electrothermal bimorph actuator design.

A bimorph is composed of two layers of different materials that have different coefficients of thermal expansion (CTEs), as shown in Figure 1a. Al and SiO_2_ are chosen as the two bimorph layers for their large CTE difference, which can lead to large actuation. A Pt resistor is also integrated as a heater. When a current is injected into the Pt resistor, Joule heating will be produced, which increases the bimorph temperature and consequently causes the bimorph to bend due to the different CTEs of the two bimorph layers. The simple bimorph shown in Figure 1a also generates undesired large lateral shift upon actuation. Thus, a bimorph actuator design consisting of two segments of silicon-backed rigid beams and three segments of Al/SiO_2_ bimorphs has been proposed, as shown in Figure 1b. This design can generate a lateral shift free (LSF) large vertical displacement [10,18].

**Figure 2:**
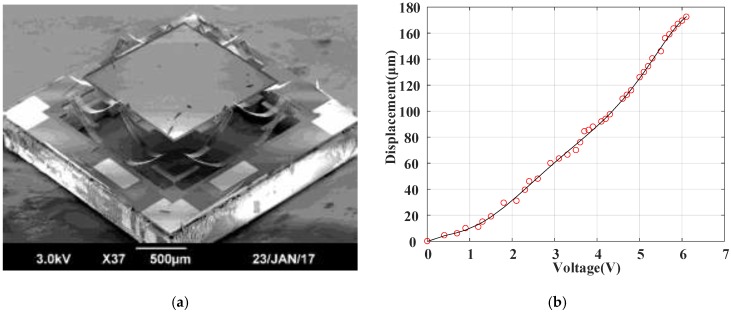
(**a**) Scanning electron microscope (SEM) of an electrothermal micro-electro-mechanical system (MEMS) mirror; (**b**) measured vertical displacement versus applied voltage on bimorph actuators.

Figure 2a shows a scanning electron microscope (SEM) of a fabricated LSF MEMS mirror, in which the initial elevation of the pop-up mirror plate is about 180 μm. This elevation is caused by the thin film intrinsic stress and thermal residual stress generated during fabrication. There are two LSF bimorph actuators attached on each side of the mirror plate. When a drive voltage is applied on all four bimorph actuators, the measured static piston response is shown in Figure 2b, in which the vertical displacement reaches up to 180 μm. The allowable drive voltage range to generate stable vertical motion is from 0 to 6.6 V. Increasing the voltage further would eventually burn out the bimorphs due to overheating. Although the optimized drive signal ratio [14] or open-loop compensation method [11] can reduce the tilting during the piston scanning, the residual tilt angle is relatively large due to time-varying characteristics of thermal bimorph actuation.

### 2.2. Dynamic Model of the Micromirror

**Figure 3:**
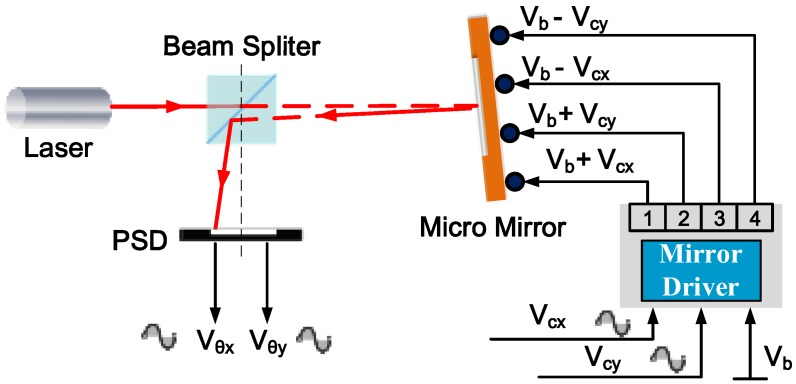
Setup to measure open-loop frequency response of micromirror tilting. PSD: position sensitive detector; *V_b_*: biased voltage; *V_cx_* and *V_cy_*: the control voltages in x and y directions; *V_θx_* and *V_θy_*: PSD output signals proportional to the mirror tilt angles in both directions of *x* and *y*.

The micromirror system studied in this paper has a complex structure and multi-input and multi-output characteristics. The analytical model is difficult to obtain. Therefore, in this work, a frequency domain experimental method is used to obtain the system model for the control system design. The frequency response of the mirror tilting motion was measured in an experimental setup as illustrated in Figure 3.

The mirror is first biased in the linear region of the scan displacement-voltage characteristics. In both directions *x* and *y*, the AC voltages *V_cx_* and *V_cy_* are superimposed on the DC bias *V_b_* via a unit-gain driver. The driver generates two pairs of the voltage outputs, *V_b_* + *V_cx_* and *V_b_* − *V_cx_*, *V_b_* + *V_cy_* and *V_b_* − *V_cy_*, which are applied to the four bimorph actuators in a differential fashion. *V_b_* is used to bias the mirror plate at a certain displacement, and *V_cx_* or *V_cy_* excites the mirror to tilt at the vertical position set by *V_b_*. To test the frequency response, the frequency of *V_cx_* and *V_cy_* are swept, and the actual tilt angles in both directions of *x* and *y* are measured by tracking the light beam reflected from the mirror plate using a position sensitive detector (PSD). The PSD output voltages, *V_θx_* and *V_θy_*, are proportional to the mirror tilt angles in both directions of *x* and *y*. 

The system has two outputs, so it needs to analyze and judge the coupling relationship. If the coupling is strong, decoupling is needed. There are a variety of methods to evaluate the degree of coupling of multivariable systems [19,20,21,22,23,24,25,26], among which the most widely used one is the static and dynamic Relative Gain Array (RGA) theory [24] proposed by Bristol.

For a multivariable control system, Bristol defines a first amplification factor *Φ_k_* and a second amplification factor *P_k_*. The first amplification coefficient *Φ_k_* means that, in the system of the mutual coupling, the channel gain between a driving signal of X or Y direction *Drv_j*(*j = x,y*) and a PSD signal of X or Y direction *PSD_i*(*i = x,y*) under the conditions of *Drv_j* observed with a change of *∆Drv_j* and other manipulated variables *Drv_r*(*r ≠ j, r = x,y*) unchanged, the $\Phi_{k}$ of *x* and *y* direction is $\Phi_{x}={\Delta PSD\_x/\Delta Drv\_x|}_{Drv\_y=const}$, $\Phi_{y}={\Delta PSD\_y/\Delta Drv\_y|}_{Drv\_x=const}$. The second amplification factor *P_k_* refers to the change of the *PSD_i*(*i = x,y*) obtained by fixing the other *PSD_r*( *r ≠ i, r = x,y*) and changing only *Drv_j*(*j = x,y*), and the two change coefficient is the second amplification factor *P_k_*. in the static state. That is, $P_{x}={\Delta PSD\_x/\Delta Drv\_x|}_{PSD\_y=const}$, $P_{y}={\Delta PSD\_y/\Delta Drv\_y|}_{PSD\_x=const}$. When $0.8<\lambda_{k}=\Phi_{k}/P_{k}<1.2$, the influence of other channels on the channel (Drv_j→PSD_i) is small and can be used as the main channel [24].

**Figure 4:**
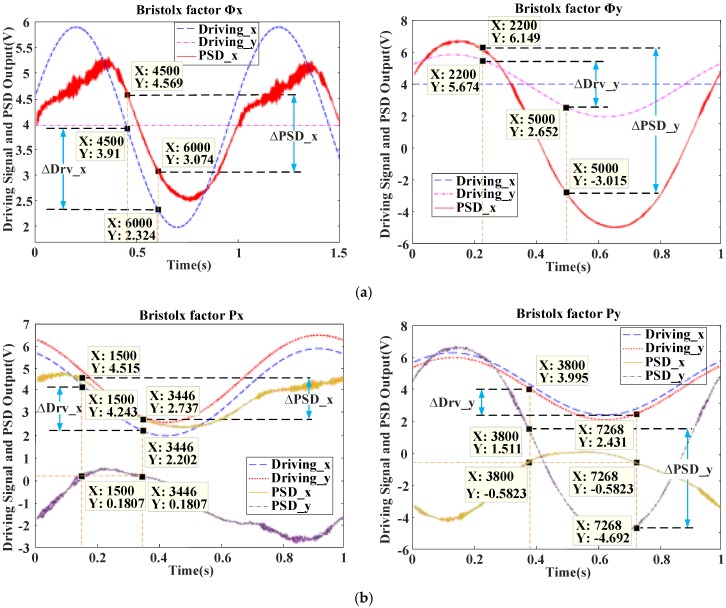
(**a**) Signals for calculating Bristolx factors *Φ* of X/Y direction; (**b**) signals for calculating Bristolx factors P of X/Y direction.

As shown in Figure 4a, X/Y direction uses 2 V~6 V sine waves to drive, and Y/X direction uses 4 V constant value to drive, get the *Φ*: *Φx* = 1.495/1.586, *Φy* = 9.164/3.022. As shown in Figure 4b, X/Y direction uses 2 V~6 V sine to drive, Y/X direction uses 2.5V ~ 6.5V sine to drive, get the *P*: *Px* = 1.778/2.041, *Py* = 1.347/0.462. Finally, the following results are obtained: (1) $\lambda_{x}=\Phi_{x}/P_{x}=1.082\in \left(0.8,1.2\right)$; (2) $\lambda_{y}=\Phi_{y}/P_{y}=1.04\in \left(0.8,1.2\right)$. *λ_x_* and *λ_y_* indicate that the control of the *x*, *y* direction can be used as the main control channel, which can independently be controlled.

As illustrated in Figure 3, the frequency responses of the x-scan and y-scan can be expressed as:

(1) $$G_{mx}\left(s\right)=\frac{V_{\theta x}\left(s\right)}{V_{cx}\left(s\right)}\;,\;G_{my}\left(s\right)=\frac{V_{\theta y}\left(s\right)}{V_{cy}\left(s\right)}$$

**Figure 5:**
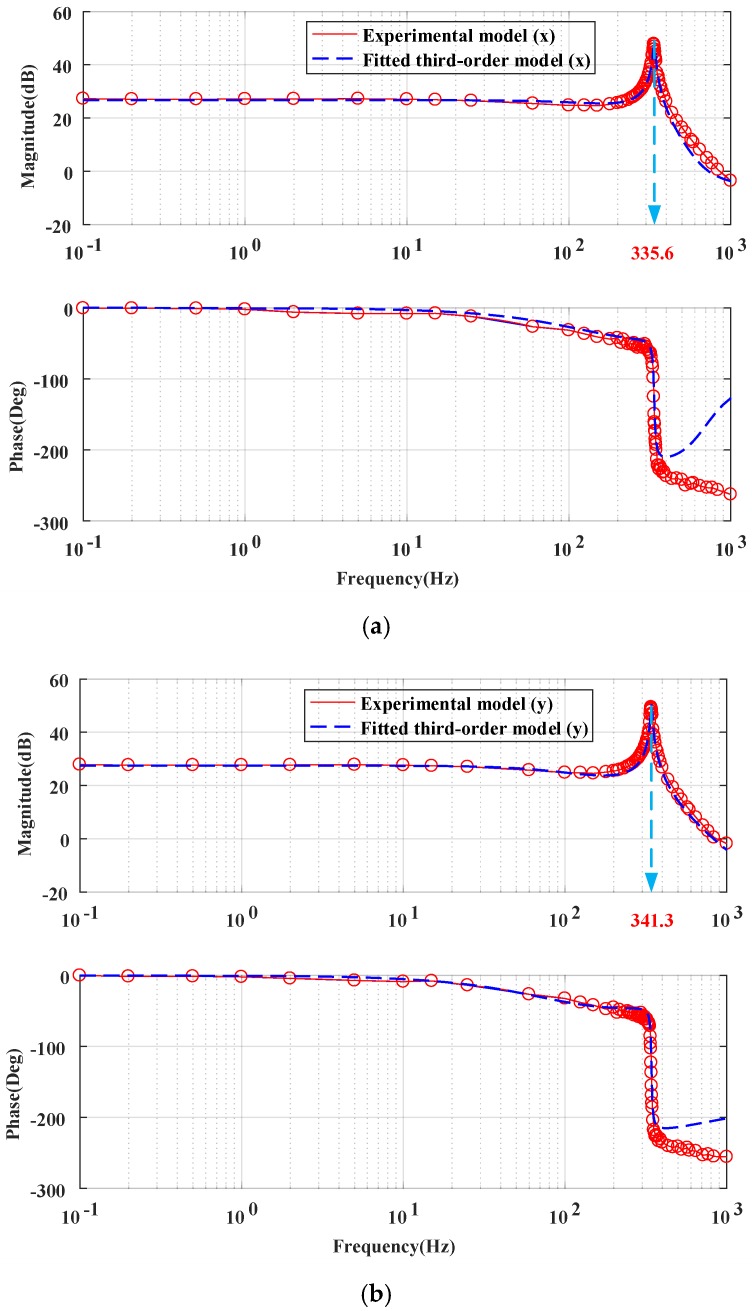
Experimental frequency response and fitted micromirror model. (**a**) The micromirror model of X direction. (**b**) The micromirror model of Y direction.

As shown in Figure 2b, the MEMS mirror has a strong nonlinear response at low voltage, but the response is quite linear from 2 V to 6 V. Thus, the MEMS mirror is typically biased at 4 V to maximize its usable linear range. The setup shown in Figure 3 is used to measure the frequency response. The PSD's photosensitive area is 10 mm × 10 mm. In order to ensure the scanning optical beam completely captured by the PSD, an ac voltage with a small amplitude of 0.1 V plus a dc bias of 4.0 V is employed. The measured frequency responses are shown in Figure 5a,b, in which the resonant frequencies in x and y direction are 335.6 Hz and 341.3 Hz, respectively.

Based on the experimental data shown in Figure 5, we used the system identification toolbox in Matlab to identify the system, and selected the best-fit results as the system model for the feedback controller design, i.e.,

(2) $$G_{mx}\left(s\right)=\frac{4915\;s^{2}+1.68\times 10^{7}\;s+9.46\times 10^{10}}{s^{3}+1042s^{2}+4.54\times 10^{6}\;s+4.36\times 10^{9}}$$

(3) $$G_{my}\left(s\right)=\frac{1.92\times 10^{7}\;s+6.16\times 10^{10}}{s^{3}+614.4s^{2}+4.66\times 10^{6}\;s+2.61\times 10^{9}}$$

This transfer function model was tested under room temperature and one atmospheric pressure. For different micromirrors or operating conditions, the model parameters can be measured and fitted by using the above identification method. Furthermore, the transfer function may change slightly when the dc bias voltage is set at a different value or the ac amplitude is increased. This effect will be investigated in the future work. 

## 3. Design of Robust Controller

When the MEMS micromirror-based FTS system is placed in different environments and operating conditions, the system parameters will change, and thus the system model will need to be changed accordingly. So, it is a great challenge to design a controller that can ensure the stability and response characteristics of the system even when external disturbances or an internal structure deterioration exist.

The PID control and look-up table control methods are simple and easy to implement, but these control methods cannot adapt well to changes in system parameters, causing large errors or even system instability. Therefore, in this work, a controller design with an H∞ hybrid sensitivity control algorithm is proposed, so that the FTS system can not only be applied to working environments other than the laboratory, but can also be adapted to the measurement errors or the parameters’ drift caused by the aging of the system. In order to evaluate the performance of the H∞ control method, a PID controller and a look-up controller are also implemented for the FTS system as a comparison.

**Figure 6:**
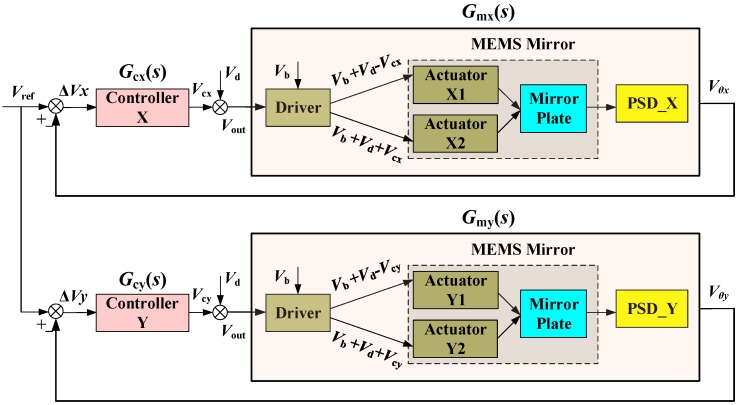
Block diagram of the closed-loop micromirror tilt control system. *G_cx_*(*s*) and *G_cy_*(*s*): the feedback controllers in x and y directions; *G_mx_*(*s*) and *G_my_*(*s*): the micromirror systems under control in x and y directions, including the drivers, actuators, micromirror plate, and position sensitive detector (PSD).

A block diagram of the micromirror tilting control loop is shown in Figure 6, in which *G_cx_*(*s*) and *G_cy_*(*s*) denote the feedback controllers to be designed, the error Δ*V_x_* and Δ*V_y_* are the measures of the residual tilting of the MEMS mirror plate, and *V*_d_ is the equivalent disturbance input voltage intended to evaluate the robustness of the closed-loop system. The system models *G_mx_*(*s*) and *G_my_*(*s*) have been experimentally obtained as given in Equations (2) and (3).

### 3.1. Design of H∞ Robust Controller

H∞ robust control theory [25] is a control theory in H∞ space (Hardy space) that can yield robust controllers by optimizing the infinite norm of certain performance indexes, solving the problems of a robust control model that has a certain range of uncertainties and external interference signals that exist in a system. 

**Figure 7:**
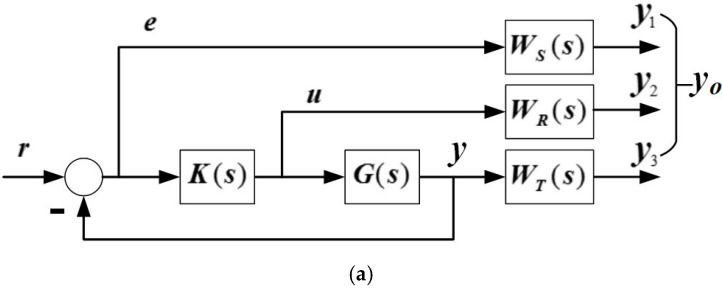

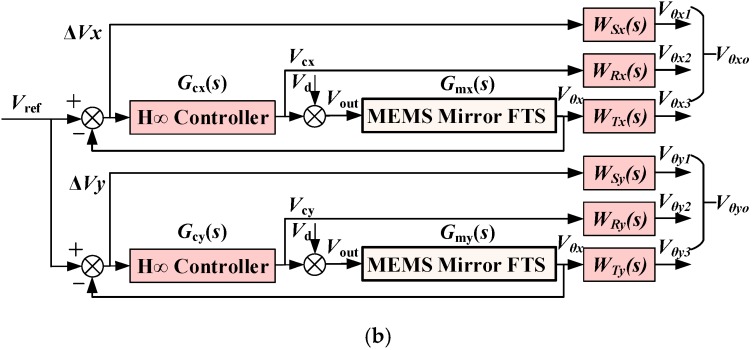
(**a**) Structure diagram of the H∞ mixed sensitivity control system. (**b**) The block diagram of the Fourier transform spectrometer (FTS) system with an H∞ controller.

To control a system with both interference and uncertainty, the H∞ mixed sensitivity design method can be employed [26]. The general control system structure diagram of the weighted H∞ mixed sensitivity method is shown in Figure 7a, in which *W_S_*(*s*), *W_R_*(*s*), and *W_T_*(*s*) are three weight functions for system quality, output control, and stability, respectively. The corresponding control block diagram of the FTS system control structure is shown in Figure 7b, in which *r* is replaced by the PSD reference input *V_ref_*, *e* by the PSD error Δ*V_x_* or Δ*V_y_*, *u* by the H∞ controller output, *V_cx_* or *V_cy_*, *y_o_* = [*y*_1_,*y*_2_,*y*_3_] by the output of the MEMS mirror in the FTS system, *V_θxo_* = [*V_θx1_*, *V_θx2_*, *V_θx3_*] or *V_θyo_* = [*V_θy1_*, *V_θy2_*, *V_θy3_*], *K*(*s*) by *G_cx_*(*s*) or *G_cy_*(*s*), *G*(*s*) by *G_mx_*(*s*) or *G_my_*(*s*), and *W_S_*(*s*), *W_R_*(*s*), and *W_T_*(*s*) *by W_Sx_*(*s*) or *W_Sy_*(*s*), *W_Rx_*(*s*) or *W_Ry_*(*s*), *W_Tx_*(*s*) or *W_Ty_*(*s*), respectively. These three pairs of weight functions are added in order to suppress the interference and reduce the uncertainty and thus improve the system performance.

According to Figure 7a, the closed-loop transfer function S(s) from the input *r* to the error *e* is given by,

(4) $$S\left(s\right)={\left[I+G\left(s\right)K\left(s\right)\right]}^{-1}$$

in which *S*(*s*) is called the sensitivity function, which is the most important indicator for determining the size of the PSD signal tracking error. The lower the sensitivity *S*(*s*) is, the smaller the tilting of the FTS system is. The closed-loop transfer function *T*(*s*) from the input *r* to the output *y_o_* is

(5) T(s)=I−S(s)

We can use the P–K structure of the H∞ standard problem. The generalized object *P*(*s*) is given by

(6) $$P\left(s\right)=\left[\begin{array}{cc} P_{11} & P_{12}\\ P_{21} & P_{22} \end{array}\right]=\left[\begin{array}{cc} \begin{array}{c} \begin{array}{c} W_{s}\\ 0\\ 0 \end{array}\\ I \end{array} & \begin{array}{c} \begin{array}{c} -W_{s}G\\ W_{R}\\ W_{T}G \end{array}\\ -G \end{array} \end{array}\right]$$

The closed-loop transfer function matrix from the input *r* to the output *y_o_* is

(7) $$T_{ry_{o}}\left(s\right)=LFT\left(P\left(s\right),K\left(s\right)\right)=P_{11}+P_{12}K{\left(I-P_{22}K\right)}^{-1}P_{21}={\left[W_{s}S,W_{R}KS,W_{T}T\right]}^{T}$$

The H∞ mixed sensitivity controller is a controller *K*(*s*) that makes the closed-loop transfer function of the FTS system internally stable and satisfies the following condition,

(8) $$\underset{K}{inf}\|T_{ry_{o}}{\left(s\right)\|}_{\infty }=\gamma_{min}\Rightarrow \|T_{ry_{o}}{\left(s)\right\|}_{\infty }<\gamma \left(\gamma >\gamma_{min}\right)$$

**Figure 8:**
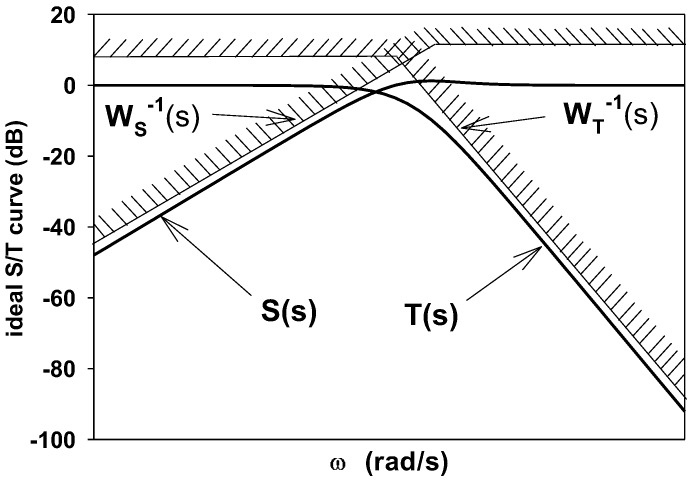
Ideal S/T curve. *S*(*s*) is the sensitivity function, *T*(*s*) is complementary sensitivity function, *W_S_*(*s*) is weight function for system quality, and *W_T_*(*s*) is weight function for stability.

From the frequency domain point of view, the classical H∞ control algorithm is essentially a system loop forming method. The H∞ mixed sensitivity control strategy directly performs closed-loop gain shaping on the closed-loop function such as the sensitivity function *S*(*s*) or the complementary sensitivity function *T*(*s*), thus eliminating the large peaks that may occur in open-loop gain shaping. The ideal *S/T* curve is given in Figure 8.

**Figure 9:**
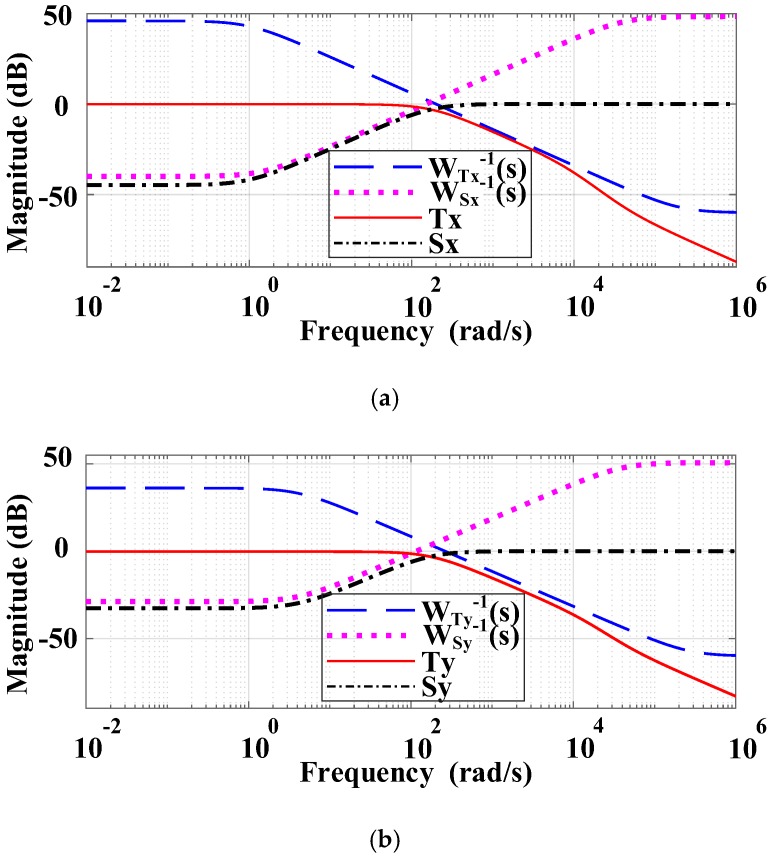
*S/T* curve. (**a**) *S/T* curve of X direction; (**b**) *S/T* curve of Y direction.

As show in Figure 9, in order to make the FTS system meet the shape of the ideal *S/T* curve, according to the FTS system characteristics, through multiple iterative tests, we set the weight functions $W_{Sx}\left(s\right)=\left(0.001s+200\right)/\left(s+1\right)$, $W_{Sy}\left(s\right)=\left(0.001s+265\right)/\left(s+4.1\right)$, $W_{Tx}\left(s\right)=\left(0.66s+1\right)/\left(0.0025s+100\right)$, and $W_{Ty}\left(s\right)=\left(0.86s+3.6\right)/\left(0.0025s+100\right)$ to guarantee the desired low-pass characteristics, and set the weight function $W_{Rx}\left(s\right)=W_{Ry}\left(s\right)=1\times 10^{-4}$ to adjust the output of the controller to ensure that the PSD output changes at the millivolt level.

Equivalently, for the system shown in Figure 7b, *K*(*s*) in Equation (8) will be simply replaced by *G_cx_*(*s*) or *G_cy_*(*s*). Thus, according to Equation (8), the optimized controller *G_cx_*(*s*) and *G_cy_*(*s*) must satisfy the following conditions,

$$\underset{G_{cx}}{inf}\|T_{ry_{o}}{\left(s)\right\|}_{\infty }=\underset{G_{cx}}{inf}{\left\|\begin{array}{c} W_{sx}S\\ W_{Rx}G_{cx}S\\ W_{Tx}T \end{array}\right\|}_{\infty }=\underset{G_{cx}}{inf}{\left\|\begin{array}{c} W_{sx}{\left(I+G_{mx}G_{cx}\right)}^{-1}\\ W_{Rx}G_{cx}{\left(I+G_{mx}G_{cx}\right)}^{-1}\\ W_{Tx}G_{mx}G_{cx}{\left(I+G_{mx}G_{cx}\right)}^{-1} \end{array}\right\|}_{\infty }\Rightarrow {\left\|\begin{array}{c} W_{sx}S\\ W_{Rx}G_{cx}S\\ W_{Tx}T \end{array}\right\|}_{\infty }<\gamma$$

$$\underset{G_{cy}}{inf}\|T_{ry_{o}}{\left(s)\right\|}_{\infty }=\underset{G_{cy}}{inf}{\left\|\begin{array}{c} W_{sy}S\\ W_{Ry}G_{cy}S\\ W_{Ty}T \end{array}\right\|}_{\infty }=\underset{G_{cy}}{inf}{\left\|\begin{array}{c} W_{sy}{\left(I+G_{mx}G_{cy}\right)}^{-1}\\ W_{Ry}G_{cy}{\left(I+G_{mx}G_{cy}\right)}^{-1}\\ W_{Ty}G_{mx}G_{cy}{\left(I+G_{mx}G_{cy}\right)}^{-1} \end{array}\right\|}_{\infty }\Rightarrow {\left\|\begin{array}{c} W_{sy}S\\ W_{Ry}G_{cy}S\\ W_{Ty}T \end{array}\right\|}_{\infty }<\gamma$$

Based on the experimental system models of *G_mx_*(*s*) and *G_my_*(*s*) and the weight functions including *W_Sx_*(*s*), *W_Sy_*(*s*), *W_Rx_*(*s*), *W_Ry_*(*s*), *W_Tx_*(*s*) and *W_Ty_*(*s*), the Matlab Robust Control Toolbox can be used to calculate the controllers that meet the above conditions.

Without loss of generality, *γ* is set to be 1. Then, the robust controllers *G_cx_*(*s*) and *G_cy_*(*s*) in the *x* and *y* directions are obtained as follows,

$$G_{cx}\left(s\right)=\frac{1.15\times 10^{8}s^{4}+4.71\times 10^{12}s^{3}+5.30\times 10^{15}s^{2}+2.13\times 10^{19}s+1.99\times 10^{22}}{s^{5}+1.29\times 10^{10}s^{4}+1.75\times 10^{14}s^{3}+6.96\times 10^{17}s^{2}+2.52\times 10^{21}s+2.52\times 10^{21}}$$

$$G_{cy}\left(s\right)=\frac{2.42\times 10^{8}s^{4}+9.81\times 10^{12}s^{3}+7.06\times 10^{15}s^{2}+4.57\times 10^{19}s+2.52\times 10^{22}}{s^{5}+1.15\times 10^{7}s^{4}+6.64\times 10^{13}s^{3}+1.29\times 10^{18}s^{2}+3.44\times 10^{21}s+1.41\times 10^{22}}$$

### 3.2. Design of the Proportional-Integral-Derivative (PID) Controller

**Figure 10:**
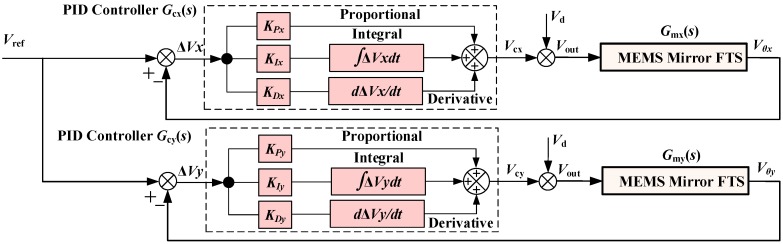
The block diagram of the proposed proportional-integral-derivative (PID) controller.

Figure 10 shows the PID controller designed to control both *x* and *y* directions. The two control loops can be independently controlled and adjusted for optimal conditions of the final controller. 

A PID controller is a kind of linear controller that minimizes the error between the reference value and the actual output value. The errors in *x* and *y* directions are given by

(9) $$\Delta V_{x}=V_{ref}-V_{\theta x},\;\Delta V_{y}=V_{ref}-V_{\theta y}$$

Its control law is

(10) $$G_{cx}\left(s\right)=\frac{V_{cx}}{\Delta V_{x}}=K_{Px}\left[1+\frac{1}{K_{Ix}s}+K_{Dx}s\right],\;G_{cy}\left(s\right)=\frac{V_{cy}}{\Delta V_{y}}=K_{Py}\left[1+\frac{1}{K_{Iy}s}+K_{Dy}s\right]$$

In the above formula, *K_P_* is the proportional coefficient, *K_I_* is the integral time constant, and *K_D_* is the differential time constant. In the PID controller of the correction link, *K_P_* adjusts the system error. Once the error occurs, the controller produces control effect to reduce the error. *K_I_* is used to eliminate the static difference and improve the system's lack of difference. *K_D_* adjusts the rate of change of the error, accelerates the speed of movement of the system, and reduces the adjustment time. 

Even without the system model, *K_P_*, *K_I_*, and *K_D_* can be experimentally determined by multiple trials. In this work, the PID controller is obtained without using the above FTS system model. The PID controllers in the *x* and *y* directions obtained by experiment and debugging are as follows:

*G_cx_*(*s*) = 0.01 × [1 + 300/*s* + 0 × *s*]

*G_cy_*(*s*) = 0.02 × [1 + 150/*s* + 0 × *s*]

### 3.3. Design of Look up Table Controller

**Figure 11:**
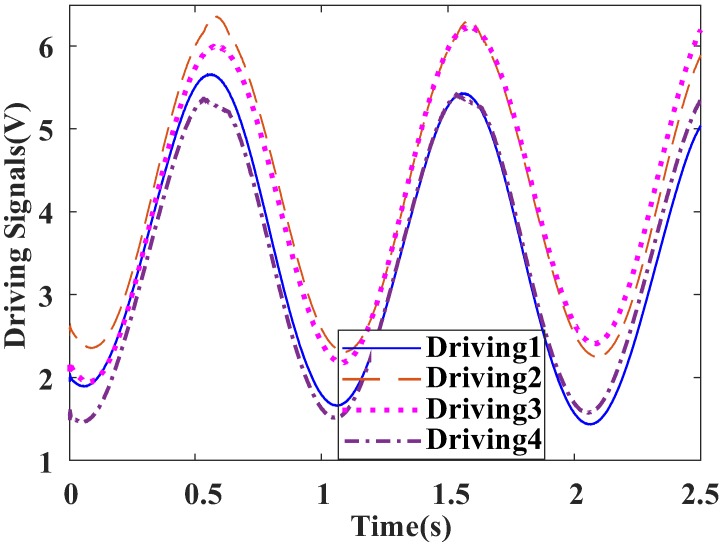
Voltage signals of four actuators under PID control.

The look-up table is actually generated from the FTS system with the PID controller described above. The output voltage signals for the four actuators X1, X2, Y1, and Y2 are acquired, and the data are plotted in Figure 11, in which *Driving1*, *Driving2*, *Driving3,* and *Driving4* are the driving voltages of the four actuators, respectively. Taking the *Driving1* as the reference, the other three signals are fitted to obtain the following results:

*Driving1* = *Driving1*; *Driving2* = 1.00045 × *Driving1* + 0.6799; *Driving3* = 0.9887 × *Driving1* + 0.6837; *Driving4* = 0.9842 × *Driving1* + 0.0038.

## 4. Experimental Results and Discussion

### 4.1. Experimental Setup

**Figure 12:**
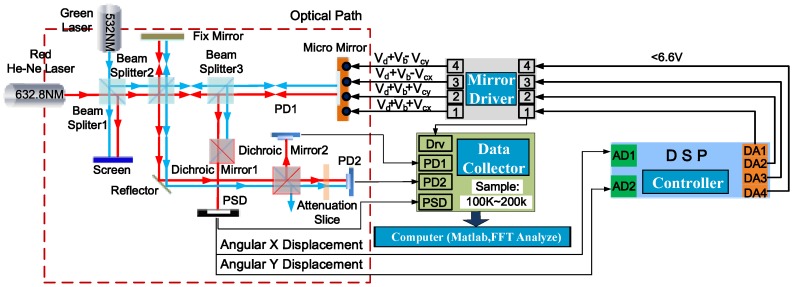
The schematic diagram of the FTS experiment system.

An experimental setup, as illustrated schematically in Figure 12, is used to evaluate the tilting performance of the closed-loop controlled electrothermal micromirror with the proposed control schemes. This setup is actually a Michelson interferometer-based Fourier transform spectrometer (FTS), which is composed of an MEMS mirror to be controlled, a red He-Ne laser (632.8 nm) source (LS-R), a green laser (532 nm) source (LS-G), three beam splitters (BS), two dichroic mirrors, a position sensitive detector (PSD), two photodiodes (PD1 and PD2), a high speed data collector, an MEMS driver to drive the four bimorph actuators in both *x* and *y* directions, and a digital controller realized with a 32-bit digital signal processor (DSP) and high speed A/D, D/A converters. Here, the controller sampling frequency is set at 10 kHz and benefits from the high-speed DSP (TMS320F28335, Texas Instruments, Texas, USA) with a powerful floating-point unit. As the frequency of the PD signals may reach 9~10 kHz, the data collector sampling rate is set to 200 kHz. A computer is used to reconstruct the spectrum via Fast Fourier Transform (FFT) from the raw interferogram signals from PD1 and PD2. 

The schematic diagram of the optical path is shown in the upper part of Figure 12. The laser beams from LS-R and LS-G are combined by the first beam splitter (BS1) and directed into the second beam splitter (BS2), in which the combined light beam is then split into two beams that are reflected back, respectively, from a fixed mirror (FM) and the MEMS mirror (MM) through the third beam splitter (BS3) and then re-combined as a single beam by the BS2. Under the combined action of the BS3 and the first dichroic mirror (DM1), the red laser light is received by the PSD. After that, the second dichroic mirror (DM2) only allows the red laser to pass through to the first photodiode (PD1) and the reflector directs the combined light to the second photodiode (PD2) and through an attenuation slice to reduce the excessive light intensity. The red laser here is introduced as the reference light for spectrum calibration to overcome the variable velocity of the mirror [14]. The green laser (532 nm) combined with the red laser is used as the testing light to be measured. As the employed MEMS micromirror has a low thermal cut-off frequency of less than 5 Hz [11,27], we use 0.2, 0.5, 1.0, and 2.0 Hz drive signals to carry out the experiments, and mainly use the results of the 1.0 Hz drive signal to perform the comparison analysis. When the drive frequency is increased, the micromirror has lower response to the high frequency drive, so that the amplitude decreases and the optical path difference (OPD) of the FTS system becomes smaller, but this is not discussed in this paper.

### 4.2. Tilting Control

The PID control, look-up table control, and H∞ control described above were, respectively, applied to the MEMS FTS system to control the tilting of the MEMS mirror under the drive signal with an amplitude of 4 V and a frequency of 1 Hz. The results are described one by one.

(1) PID control

**Figure 13:**
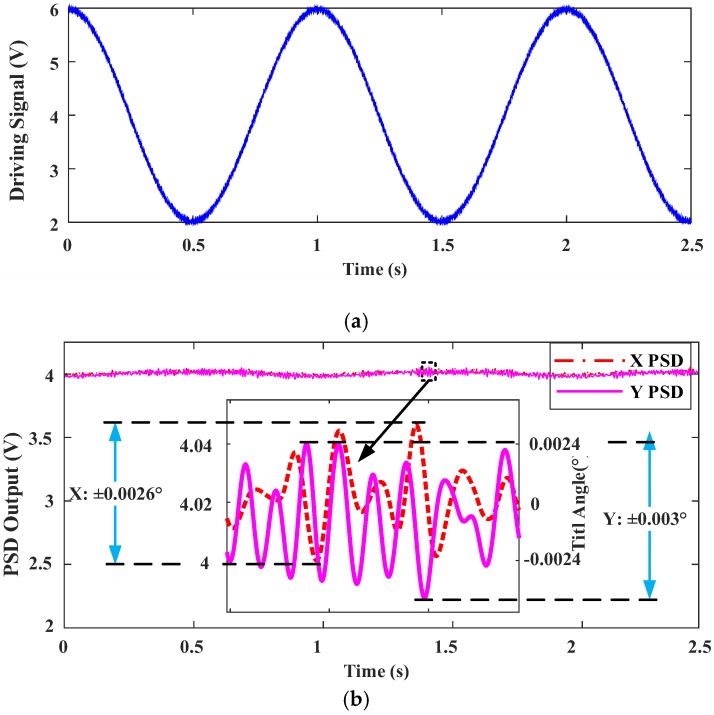
Measured tilt angle responses for PID FTS system. (**a**) Driving Signal. (**b**) PSD signals of X and Y two directions.

Using the PID controller design shown in Figure 10 and tuning *KP*, *KI*, and *KD*, we used the driving signal shown in Figure 13a and obtained the PSD output signals of the *x* and *y* two directions as shown in Figure 13b, in which the tilt angle in *x* direction was reduced to ±0.0026° from 0.453° when no control was used, and the tilt angle in *y* direction was reduced to ±0.003° from 1.786° when no control was used, both close to the optimal tilt angle range.

When changing the drive signal frequency, the tilt angles in both *x* and *y* directions vary slightly, as shown in Table 1. As the PID parameters are optimized for the 1 Hz, 2 V~6 V sine wave drive signal, the tilting increases due to the limited robustness of the PID controller when using other drive frequencies.

(2) Look-up table control

**Figure 14:**
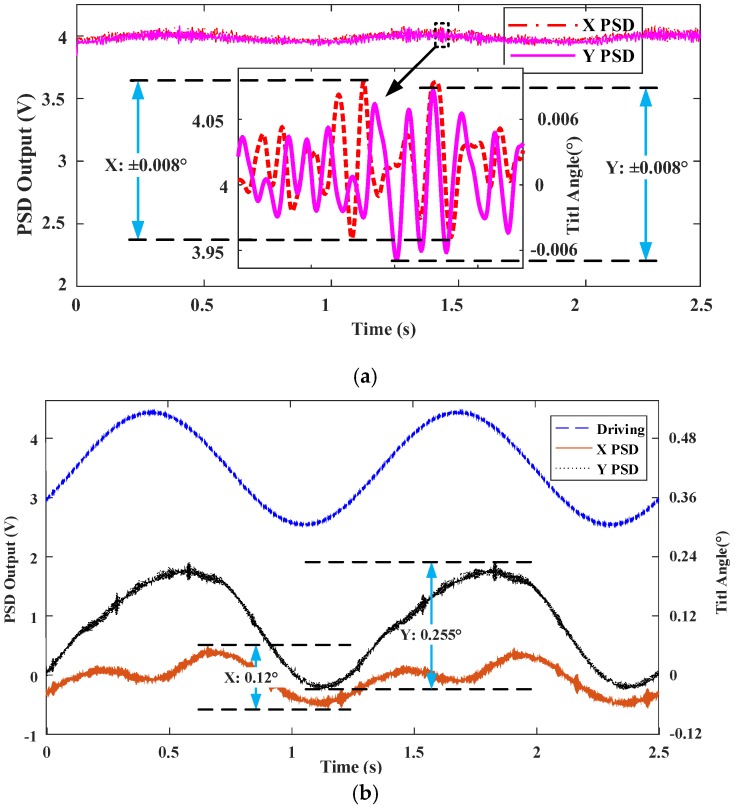
(**a**) Measured tilt angle responses for Look-up Table FTS system using the same driving as PID control. (**b**) Measured tilt angle responses for Look-up Table FTS system using different driving.

The first drive signal is set as a 1 Hz, 2 V~6 V sinusoidal signal, as shown in Figure 13a, and the other three drive signals are generated according to the method described in Section 3.3. The corresponding output signals of the PSD in *x* and *y* directions are shown in Figure 14a, in which the tilt angles in *x* and *y* directions both are ±0.008°, which are greatly reduced compared to the no-control case. However, the robustness of the look-up table control method is poor. As shown in Figure 14b, when the drive voltage is changed to a 1Hz, 2.5 V~4.5 V sine wave, the tilt angles in *x* and *y* directions are increased to ±0.12° and ±0.255°, respectively.

(3) H∞ robust Control

**Figure 15:**
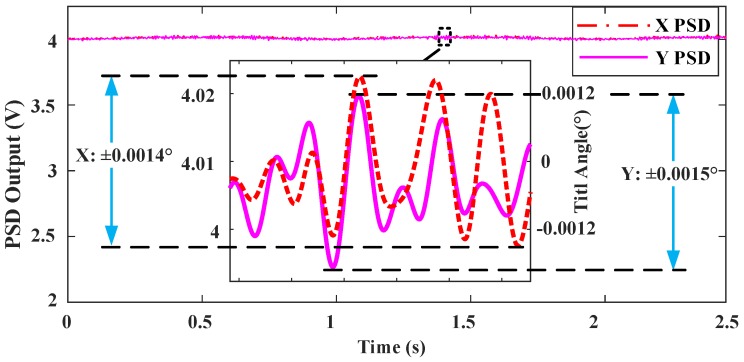
Measured tilt angle responses for FTS system with H∞ control.

The H∞ controller design given in Section 3.1 is applied to control the MEMS mirror in the FTS system with a 1 Hz, 2 V~6 V sine drive signal, as shown in Figure 13a. The tilt angles of the *x* and *y* direction are reduced down to ±0.0014° and ±0.0015°, respectively, as shown in Figure 15, which are within the optimal tilt angle range. When the frequency of the drive signal is changed to 0.2 Hz, 0.5 Hz, or 2 Hz, the maximum tilt angles are all smaller than 0.0018°. This result indicates the robustness of the H∞ controller.

### 4.3. Spectral Measurement Experiments Using the H∞ controled Fourier-Transform Spectrometer

**Figure 16:**
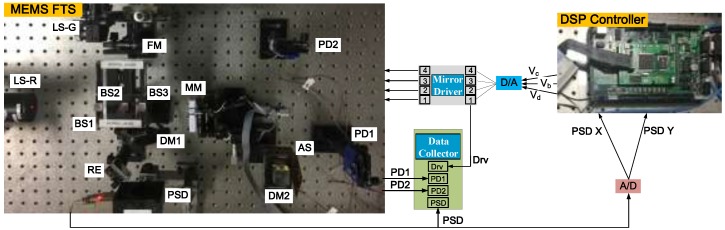
Experimental setup for MEMS FTS control system.

The FTS system as illustrated in Figure 12 has been built with the MEMS mirror. Figure 16 shows a picture of the implemented system. The interferograms of the reference light and the unknown light are picked up concomitantly by two photodetectors, PD1 and PD2, and then digitized by a data acquisition module. The usable OPD scan range is only a fraction of the total MEMS mirror scan range. Here, under the sinusoidal drive of 1 Hz and 2 V~6 V, the OPD of the micromirror is 221.8 μm. Theoretically, the spectral resolution is inversely proportional to the usable OPD scan range in which the amplitude of the interferogram fringes does not have significant loss [1]. Then, the Piecewise Cubic Hermite Interpolating Polynomial (PCHIP) interpolation is employed to convert the testing light interferogram data into an evenly sampled interferogram data of the testing light in the spatial domain. After that, the spatial domain interferogram is transformed into a spectrogram via FFT and Mertz phase correction [28]. The corresponding spectrum of the testing light source can be recovered finally.

In the following, the experimental results of the FTS system with the MEMS under respective look-up table control, PID control, and H∞ control are compared and analyzed. In all four cases, the MEMS was driven by a 1 Hz and 2 V~6 V sinusoidal voltage signal.

(1) Look-up table control

**Figure 17:**
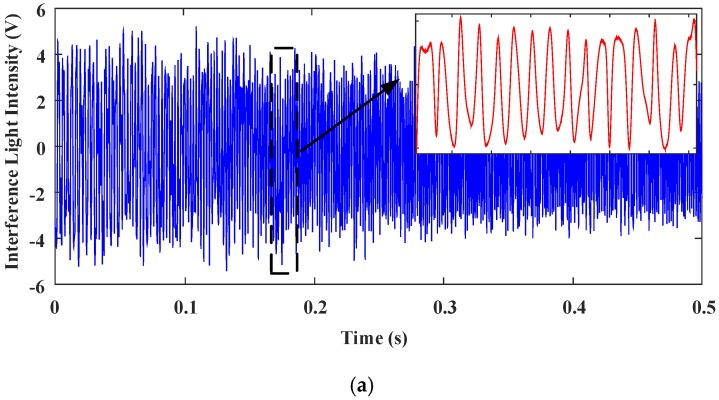

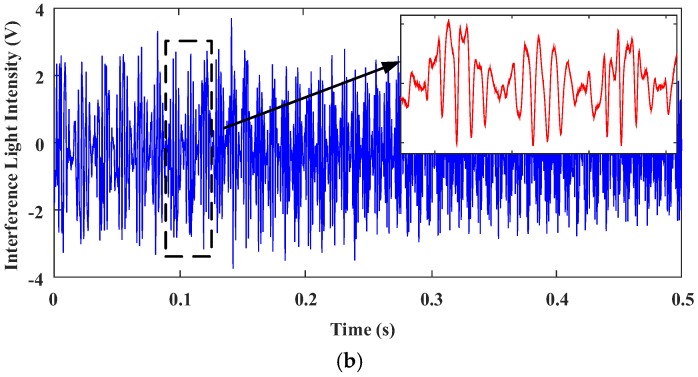
The interferogram signals acquired in time domain with look-up table control using the same driving as PID control. (**a**) Reference light. (**b**) Testing light.

Figure 17 shows the interferograms of the reference light and the testing light when a look-up table control was applied to the MEMS mirror. As the look-up table control is not robust, the quality of the interferogram signals under the look-up table control is slightly worse than the PID control, and there is some burr noise.

**Figure 18:**
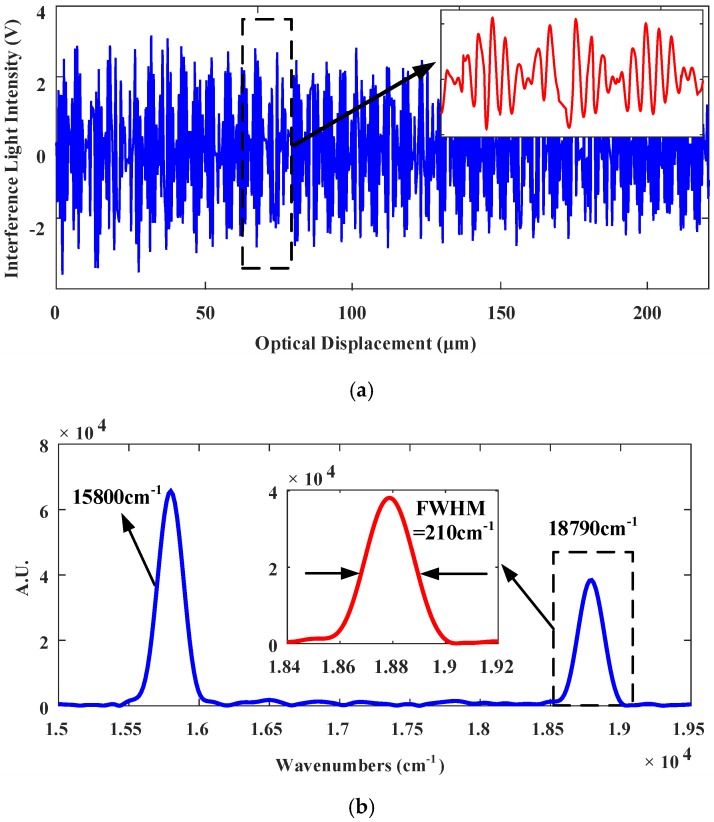
Under look-up table control using the same driving as PID control (**a**) The reconstructed interferogram of the testing light in spatial domain testing light. (**b**) Spectrum recovered of the testing light.

Figure 18a shows the reconstructed interferogram of the testing light in spatial domain with look-up table control and the corresponding spectrum. Figure 18b shows the spectrum recovered of the testing light in the system with Look-up table control. The measured full-width at half-maximum (FWHM) resolution is 210 cm^−1^, corresponding to 5.94 nm; error is slightly larger.

**Figure 19:**
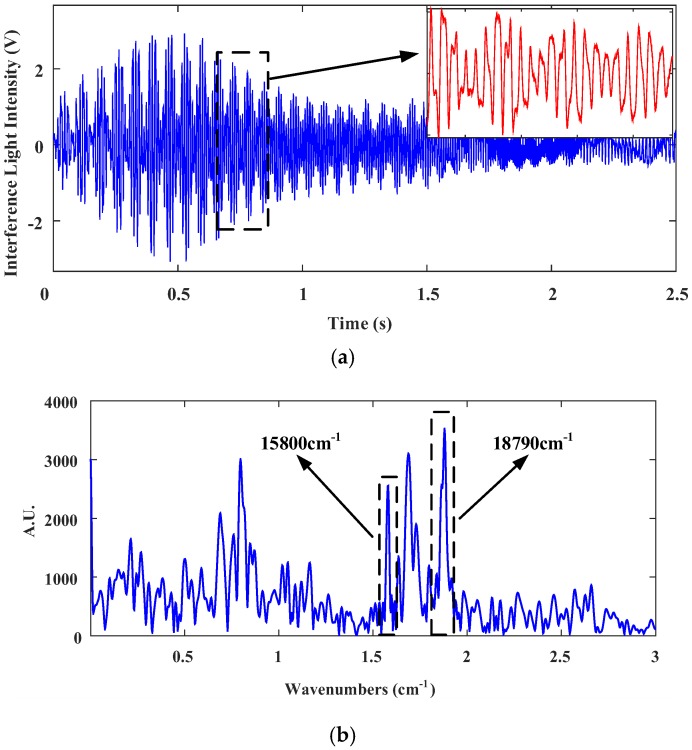
Under look-up table control using different driving (**a**) The interferogram signals of testing light acquired in time domain. (**b**) Spectrum recovered of the testing light.

When the drive signal is 1 Hz, 2.5 V~4.5 V sinusoidal drive, as shown in Figure 19, tilting is increased a lot, and the noise is also greatly increased. The resulting spectral quality is very poor, and the spectrum of the test laser is mixed in the noise and is difficult to distinguish.

(2) PID control

**Figure 20:**
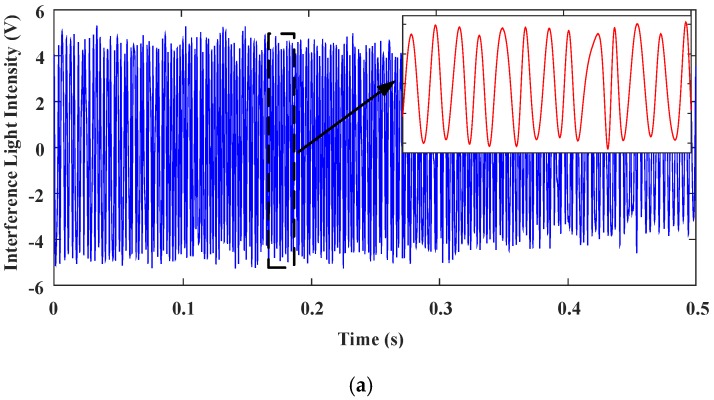

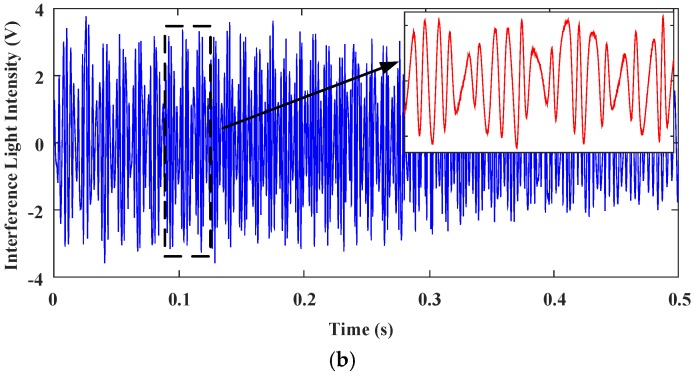
The interferogram signals acquired in time domain with PID control. (**a**) Reference light. (**b**) Testing light.

Figure 20 shows the interferograms of the reference light and the testing light, acquired when the MEMS mirror was under PID control. The quality of the interferogram signals under PID control is improved significantly. The distortion of the interferograms and the loss of the fringe contrasts are much reduced. The envelope of the reference interferogram signal has only small variations, indicating that there is only a small residual titling of the MEMS mirror left in its full scan.

**Figure 21:**
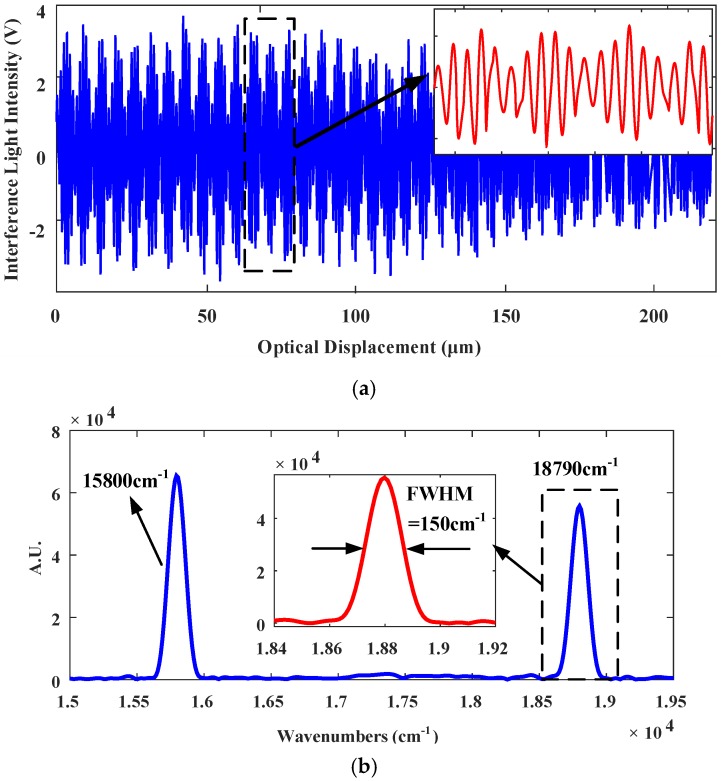
Under PID control: (**a**) the reconstructed interferogram of the testing light in spatial domain testing light; (**b**) spectrum recovered of the testing light.

Figure 21 shows the reconstructed interferogram in spatial domain of the testing light and its corresponding spectrum after FFT. It is obvious that under PID control the quality of the spectrum is greatly improved. The spectral peaks of the testing light, which is a combination of the He–Ne laser and the green laser, are detected accurately at 15,800 cm^−1^ and 18,790 cm^−1^, or 632.9 nm and 532.2 nm in wavelength, respectively. As shown in Figure 21b, the measured FWHM resolution is 150 cm^−1^, corresponding to 4.24 nm at 532 nm wavelength. Since the Gaussian window is employed for apodization, the theoretical value of the FWHM resolution is given by 2.0/OPD [1] or 90.17 cm^−1^ for this FTS, corresponding to 2.55 nm at 532 nm, as the OPD is 221.8 μm. This deviation is believed to be caused mainly by the residual tilting of the MEMS mirror.

(3) H∞ robust control

**Figure 22:**
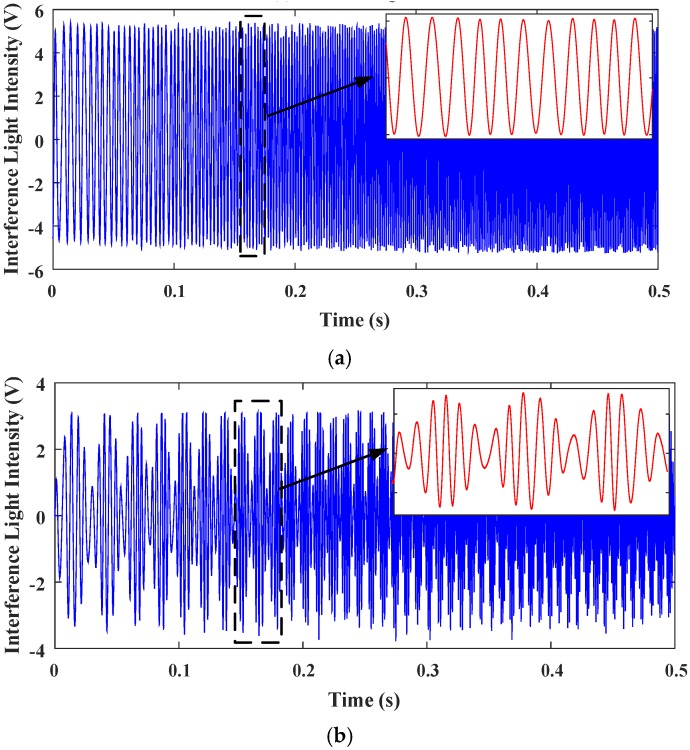
The interferogram signals acquired in time domain with H∞ control. (**a**) Reference light. (**b**) Testing light.

**Figure 23:**
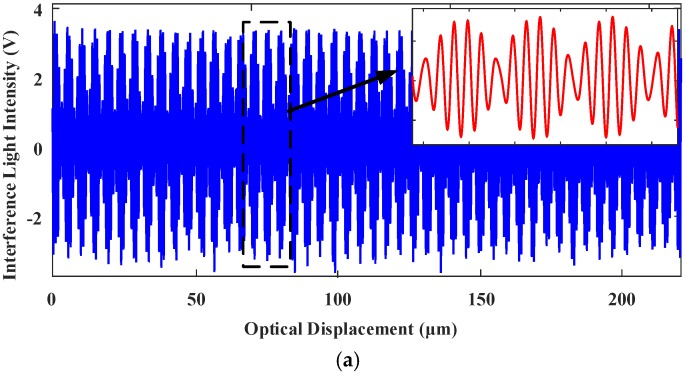

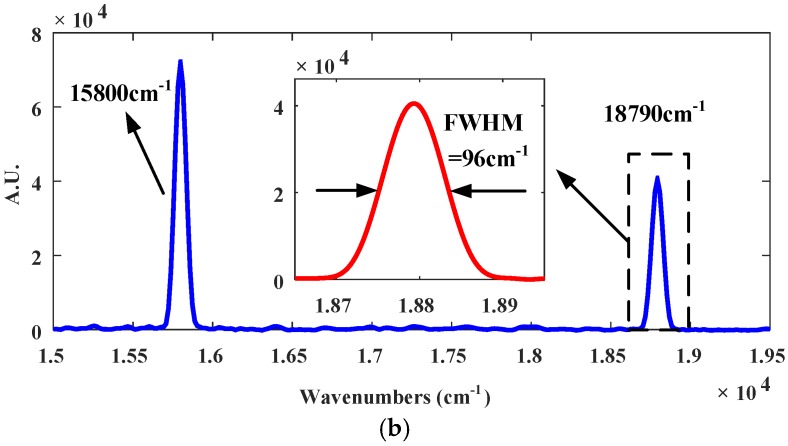
Under H∞ control: (**a**) the reconstructed interferogram of the testing light in spatial domain testing light; (**b**) spectrum recovered of the testing light.

Figure 22 shows the interferograms of the reference light and the testing light when the H∞ control was applied to the MEMS mirror. Figure 23 shows the reconstructed interferogram of the testing light in spatial domain with H∞ control and the corresponding spectrum. Under the H∞ control, the measured FWHM resolution is 96 cm^−1^, corresponding to a spectral resolution 2.71 nm at 532.2 nm, which is in good agreement with the theoretical calculation. By changing the frequency of the drive signal to 0.2, 0.5, and 2.0 Hz, the reconstructed spectra and measured FWHM’s are basically the same as those driven at 1.0 Hz, which further proves that the designed H∞ robust controller is robust.

## 5. Conclusions

In this paper, the methods of controlling the tilting of the MEMS mirror in an MEMS-based Fourier transform spectrometer are studied and experimentally verified. Based on the study of the MEMS mirror’s biaxial coupling relationship, an H∞ mixed sensitivity controller is designed to suppress the tilting of the mirror plate in both *x* and *y* directions for the purpose of maintaining pure piston motion. Compared with the PID control and look-up table control, the H∞ control has better robustness. Experimental results demonstrate that the residual tilting is as small as 0.0014° under the H∞ control. In the built MEMS FTS system, the OPD generated by the MEMS mirror reaches 221.8 μm and a spectral resolution of 96 cm^−1^, or 2.71 nm at 532 nm, has been achieved. Compared with the previous work, the mirror tilting is reduced dramatically by the H∞ control; the robustness and anti-interference capability of the FTS system are also improved.

Furthermore, considering that the LSF bimorph-based MEMS mirror employed in this study has a large thermal response time (~100 ms), which will limit the scan speed, our future work will focus on the design, fabrication, and control of a large-stroke electrothermal micromirror with a faster thermal response [29].

## Figures and Tables

**Table 1:** Tilt angle variations when the drive signal frequency changes.

Tilting Angle	Drive Signal Frequency (Hz)			
	0.2	0.5	1	2
X direction	±0.0028°	±0.0029°	±0.0026°	±0.0029°
Y direction	±0.0031°	±0.0033°	±0.003°	±0.0035°
